# Cardiovascular risk factors and carotid plaque components in a multi-ethnic cohort using 3 Tesla MRI: the HELIUS study

**DOI:** 10.1007/s00330-026-12713-2

**Published:** 2026-06-27

**Authors:** Esther M. C. Vriend, Thomas A. Bouwmeester, Daniel Bos, Didier Collard, Eric P. Moll van Charante, Henrike Galenkamp, Renske Merton, Aart J. Nederveen, Pim van Ooij, Oscar H. Franco, Bert-Jan H. van den Born

**Affiliations:** 1 Amsterdam UMC, University of Amsterdam, Department of Internal Medicine, Section Vascular Medicine, Amsterdam Cardiovascular Sciences, Amsterdam, The Netherlands; 2 Amsterdam UMC, University of Amsterdam, Department of Public and Occupational Health, Amsterdam Public Health Research Institute, Amsterdam, The Netherlands; 3 Department of Radiology and Nuclear Medicine, Erasmus MC, Rotterdam, The Netherlands; 4 Department of Epidemiology, Erasmus MC, Rotterdam, The Netherlands; 5 Department of General Practice, Amsterdam UMC, University of Amsterdam, Amsterdam, The Netherlands; 6 Department of Radiology and Nuclear Medicine, Academic Medical Center (AMC), University of Amsterdam, Amsterdam, The Netherlands; 7 Julius Center for Health Sciences and Primary Care, University Medical Center Utrecht, Utrecht University, Utrecht, The Netherlands

**Keywords:** MRI, Carotid plaques, Cardiovascular risk factors, Cardiovascular disease, HELIUS study

## Abstract

**Objective:**

Carotid plaque composition, including calcifications, lipid-rich necrotic core (LRNC), and intraplaque haemorrhage (IPH), is associated with an increased risk of cardiovascular disease (CVD). Its association with cardiovascular risk factors remains unclear, particularly in midlife and across ethnic groups. We examined the relation between cardiovascular risk factors and carotid plaque components using MRI.

**Materials and methods:**

We conducted 3-T MRI of the carotid arteries in 356 Dutch, South-Asian Surinamese, and Moroccan participants from the HELIUS study. Median follow-up time between the cardiovascular risk factor visit and MRI was 8.4 years. Multivariable logistic mixed regression models assessed associations between cardiovascular risk factors and plaque components

**Results:**

At baseline, participants had a median age of 54.0 years [IQR: 48.0–59.0] with 42.1% women. Plaque calcifications, LRNC, and IPH were present in 62.6%, 21.3%, and 3.9% of participants, respectively. Hypertension was strongly associated with calcifications (OR 4.02, 95% CI: 1.59–10.12), while smoking was both related to the presence of calcifications (OR 4.94, 95% CI: 1.79–13.62) and LRNC (OR 2.88, 95% CI: 1.30–6.40). The significant associations between both history of CVD and diabetes with calcifications were attenuated after adjusting for other risk factors. No significant associations were found for IPH, likely due to its low prevalence. There were no differences in plaque component prevalence between South-Asian Surinamese and Dutch, while Moroccans had a lower prevalence of calcifications.

**Conclusions:**

Unfavourable cardiovascular risk profiles in midlife were associated with an increased prevalence of carotid plaque components later in life, with ethnic differences in their prevalence.

**Key Points:**

***Question*** *There is limited understanding of how cardiovascular risk factors in midlife are associated with carotid plaque components across ethnicities, hampering targeted prevention of CVD*.

***Findings*** *Hypertension and smoking were strongly associated with carotid plaque calcifications; smoking was associated with LRNCs, while for IPH, no associations were found*.

***Clinical relevance*** *Unfavourable cardiovascular risk profiles in midlife increase the prevalence of carotid plaque components later in life, with ethnic differences in their prevalence, highlighting opportunities for personalised, targeted prevention strategies to reduce CVD*.

**Graphical Abstract:**

**Figure:**
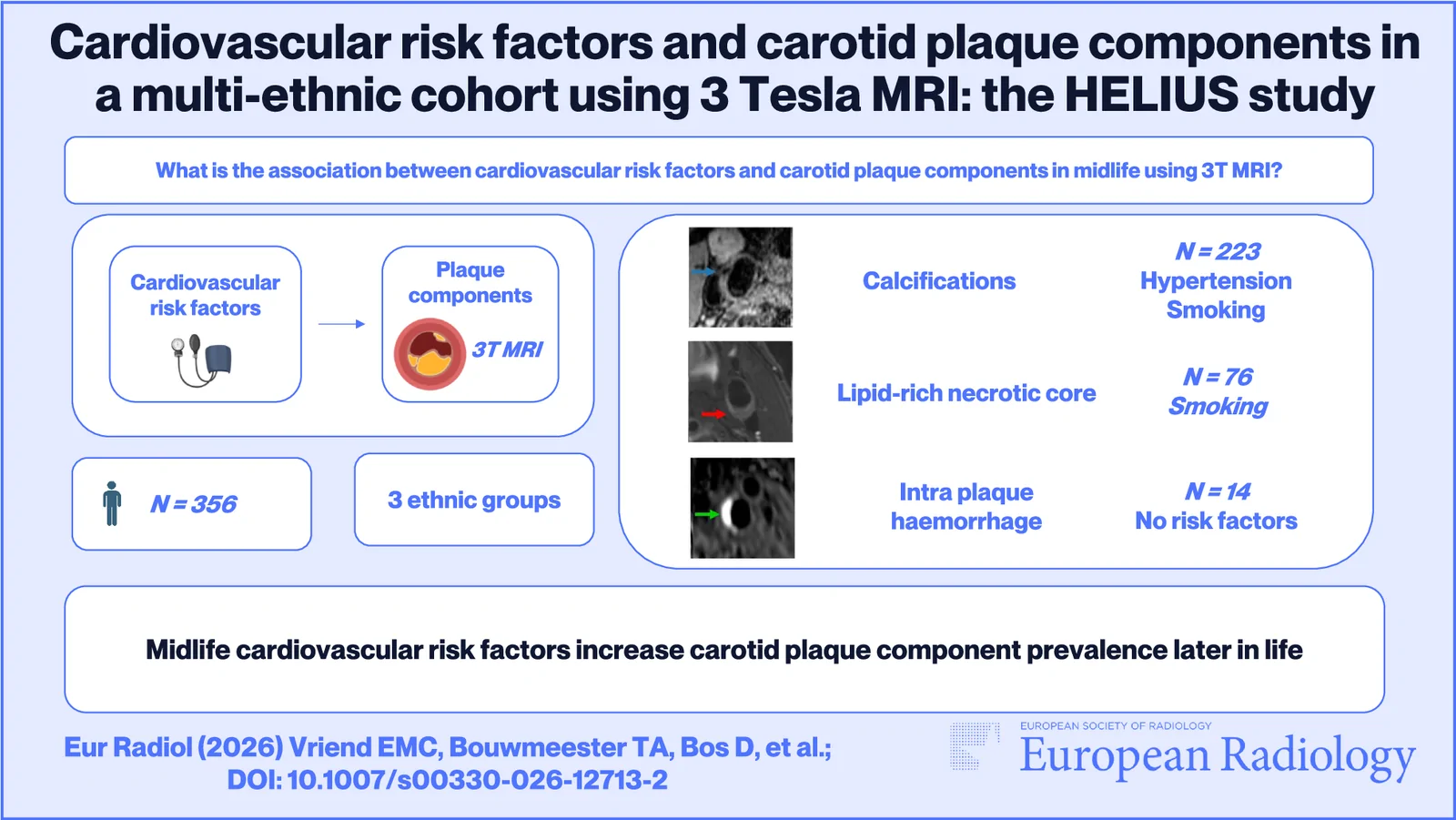

## Introduction

In recent years, the composition of carotid atherosclerotic plaques has gained rapid interest as a potential indicator of plaque vulnerability and risk of subsequent ischaemic events [1–3]. The presence of a lipid-rich necrotic core (LRNC) or intraplaque haemorrhage (IPH) are important marker of plaque vulnerability, while the presence of calcification may be indicative of more stable plaques [4, 5]. Current knowledge on the determinants of plaque components remains limited to studies that investigated cardiovascular risk factors and plaque components in patients with symptomatic carotid artery stenosis or the elderly population [6, 7]. However, from a population-based perspective, studies on the association of cardiovascular risk factors in mid-life participants with plaque composition are relatively scarce. Existing population-based studies have primarily included older participants of white European descent [8–12]. There is a lack of studies focusing on midlife populations that incorporate multi-ethnic cohorts and include a long interval between risk factor assessment and plaque characterisation. This knowledge is essential, as it may provide the foundation for earlier preventive interventions in relatively healthy individuals.

In this large, multi-ethnic, population-based cohort study, we aim to identify the associations between cardiovascular risk factors and the presence of calcification, LRNC, and IPH in midlife participants using cardiovascular risk factor data measured 8.4 years prior to the magnetic resonance imaging (MRI) assessment.

## Materials and methods

### Study setting

The current study was embedded in the HELIUS study, which is performed in compliance with the Declaration of Helsinki and has been approved by the Amsterdam UMC, location AMC institutional review board. Written informed consent was provided by all participants before enrolment in the study. The HELIUS study is a prospective, multi-ethnic, population-based cohort study conducted in Amsterdam, the Netherlands. Details of the HELIUS study have been published previously [13]. Participants were randomly invited through the municipal registry, stratified by ethnic group. For the current analysis, we included 356 participants with cardiovascular risk factors assessed between 2011 and 2015 and carotid 3 T MRI performed between 2022 and 2023 (median follow-up 8.4 years [interquartile range (IQR): 7.4–9.5]). MRI participants were selected from a HELIUS sub-study in which 3,012 Dutch, Moroccan, or South-Asian Surinamese participants underwent carotid ultrasonography (US) between 2021 and 2022 to assess plaques and carotid intima-media thickness (cIMT) [14]. Ten per cent of the US sub-study were randomly selected as a reference group (*n* = 300), while the remainder were invited based on atherosclerosis detected on US (case group, *n* = 271; cIMT ≥ 1.0 mm or plaques ≥ 2.5 mm) (Supplementary Fig. 1). After excluding 36 participants due to incomplete or poor-quality MRI, 535 participants remained, of whom 356 had plaques in one or both carotid arteries and were included in the final analyses (Supplementary Fig. 2).

### MRI scan protocol

The MRI examination was conducted according to standardised protocols using a 3 Tesla Philips Ingenia MRI scanner equipped with bilateral phased array surface coils. Initially, the carotid arteries were identified using 2D phase contrast angiography (PCA). Subsequently, the carotid arteries were visualised using an isotropic coronal black blood (BB) T1-weighted improved motion-sensitised driven-equilibrium imaging sequence (repetition time (TR) = 10 ms, echo time (TE) = 3.6 ms, flip angle = 6^◦^, acquired voxel size = 0.50 × 0.50 × 0.50 mm^3^). To detect IPH, the Simultaneous Non-contrast Angiography and intraPlaque haemorrhage (SNAP) sequence was acquired in a coronal plane (TR = 10 ms, TE = 5 ms, flip angle = 11^◦^, acquired voxel size = 0.80 × 0.80 × 1.60 mm^3^) [15]. We used the SNAP sequence because it allows simultaneous non-contrast angiography and sensitive detection of IPH, providing accurate IPH visualisation in a single efficient scan [15]. Finally, transverse 2D BB T2 scans (TR = 3000 ms, TE = 80 ms, flip angle = 90°, in-plane resolution = 0.55 × 0.76 mm^2^, slice thickness = 3.00 mm) were acquired perpendicular to the vessel and focused on the thickest segment of plaque, if present. An overview of the MRI sequence parameters can be found in Supplementary Table 1.

### Image review and reliability measures

**Fig. 1:**
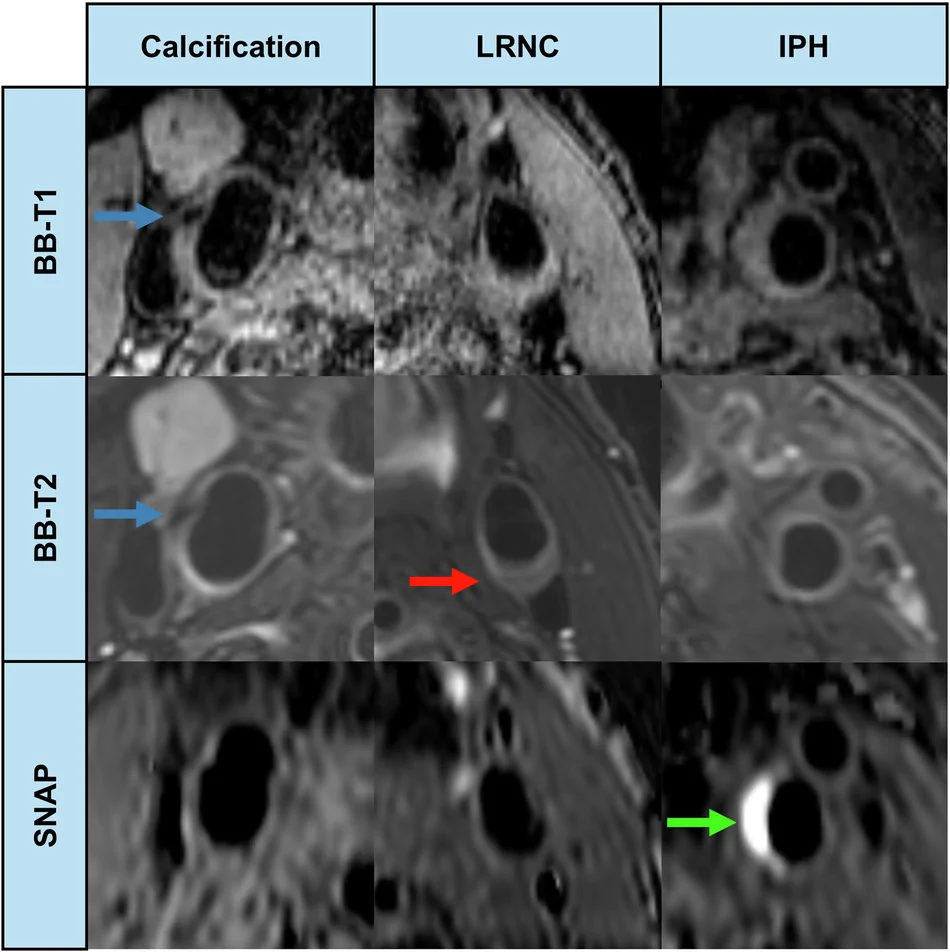
Typical example of a plaque with calcification, LRNC and IPH. Calcifications are hypointense on BB-T1-weighted and BB-T2-weighted images (blue arrow). LRNC is isointense on BB-T1-weighted images with a signal drop on BB-T2-weighted images (red arrow). IPH is isointense on BB-T1-weighted and BB-T2-weighted images and hyperintense on the SNAP sequence (green arrow). BB, black blood; IPH, intraplaque haemorrhage; LRNC, lipid-rich necrotic core; SNAP, simultaneous non-contrast angiography

Image analysis was performed using RadiAnt DICOM Viewer by two readers blinded to participant characteristics. Discrepancies were resolved by a third reader, under the supervision of an experienced physician (D.B.). Image quality was determined by two readers or by the third reader in case of disagreement [8, 16]. The degree of stenosis was calculated using the NASCET criteria [17]. Plaque characteristics, including calcification, LRNC, and IPH, were assessed in the common carotid artery, bifurcation, and internal carotid artery using BB-T1, BB-T2, and SNAP sequences. Three different plaque characteristics were assessed: calcification (hypointense on BB-T1-weighted and BB-T2-weighted images), LRNC (isointense on BB-T1-weighted images and signal drop on BB-T2-weighted images), and IPH (isointense on BB-T1-weighted and BB-T2-weighted images, hyperintense on SNAP sequence) (Fig. 1).

A random subsample of scans (*n* = 60) was read in duplicate by the same assessor to calculate intra-observer variability. Since IPH was not present in this subset, measures for IPH could not be calculated. Cohen’s Kappa for plaque presence, calcification, and LRNC showed an excellent correlation: 0.96 (95% CI: 0.87–1.0) for plaque presence, 0.93 (95% CI: 0.83–1.0) for calcification, and 0.89 (95% CI: 0.74–1.0) for LRNC. The intraclass correlation coefficient (ICC) for plaque thickness was 0.95 (95% CI: 0.88–0.98).

As all scans were read by two independent assessors, the calculation of inter-observer variability could be performed with data from 535 participants. Cohen’s Kappa for plaque presence, calcification, and IPH also showed excellent correlation: 0.89 (95% CI: 0.84–0.93) for plaque presence, 0.90 (95% CI: 0.86–0.94) for calcification, and 0.84 (95% CI: 0.69–0.99) for IPH, while the inter-reader reproducibility for the LRNC was modest (0.66, 95% CI: 0.56–0.75). The ICC for plaque thickness was 0.75 (95% CI: 0.69–0.80).

### Cardiovascular risk factor assessment

Fasting plasma samples were used to measure creatinine, total cholesterol, low-density lipoprotein (LDL) and high-density lipoprotein (HDL) cholesterol, lipoprotein(a), and glucose. Medication use was recorded and coded according to the Anatomical Therapeutic Chemical (ATC) system. Body mass index (BMI) was calculated as weight (kg) divided by height (m²). Blood pressure (BP) was measured twice on the left arm after ≥ 5 min of rest using a validated semi-automatic device, with the average used for analysis. Hypertension was defined as BP ≥ 140/90 mmHg or use of antihypertensive medication. Diabetes mellitus was defined as fasting glucose ≥ 7 mmol/L or use of glucose-lowering medication. eGFR was calculated using the CKD-EPI 2021 creatinine equation without race correction, and chronic kidney disease (CKD) was defined as eGFR < 60 mL/min/1.73 m² and/or albumin–creatinine ratio ≥ 3 mg/mmol. The history of CVD included self-reported stroke, myocardial infarction, or coronary/peripheral revascularisation. Smoking status was categorised as current, former, or never smokers.

### Statistical analyses

Descriptive statistics were presented as mean (standard deviation (SD)), median [IQR], or *n* (%). Scatterplots visualised the relation between age and plaque thickness, with calcification, LRNC, and IPH indicated in different colours. Multivariable logistic mixed regression models were constructed for each plaque component separately, including a random effect to account for correlation between left and right carotid arteries. Model 1 assessed each cardiovascular risk factor individually, adjusting for age at baseline, sex, ethnicity, inclusion group (0 = reference, 1 = case), follow-up time, and maximum wall thickness. Assessed risk factors included BMI, smoking, diabetes, total cholesterol/HDL ratio, hypertension, CKD, and CVD history. Model 2 included all risk factors simultaneously with the same covariates. An analysis using inverse probability weighting to adjust for the study design was also conducted, and the results were not materially different (data not shown).

Four sensitivity analyses were performed: excluding participants with baseline CVD, varying plaque component combinations, adjusting for medication use (antithrombotic, lipid-lowering and antihypertensive), and substituting hypertension with SBP/DBP and total cholesterol/HDL ratio with lipoprotein(a) or adjusted LDL levels (no significant associations observed). LDL levels were adjusted for the use of medication following previous publications [18, 19]. Statistical significance was set at *p* < 0.05, and analyses were conducted in RStudio (v4.3.2).

## Results

### Characteristics of the included population

At the baseline visit, the median age was 54.0 years [IQR: 48.0–59.0], with 42.1% being women (Table 1). The majority was of Dutch descent (48.0%), followed by individuals of South-Asian Surinamese (33.4%) and Moroccan (18.5%) descent. Hypertension was present in 40.5% of the sample, and 8.0% had a positive history of CVD. Among participants with specific plaque characteristics, those with IPH were the oldest (median age 60.5 years [IQR: 58–63.8]), and 14.3% were women. Use of antithrombotic medication was more common in all plaque component subgroups compared to the total population, except for the group with IPH (7.1%). The prevalence of CVD history was highest in participants with IPH (14.3%), followed by those with calcifications (11.3%) and LRNC (9.3%).

**Table 1:** Characteristics of the study population at baseline

	All participants *n* = 356	Participants with calcifications *n* = 223, 62.6% of all participants	Participants with LRNC *n* = 76, 21.3% of all participants	Participants with IPH *n* = 14 3.9% of all participants	Participants without plaque characteristics *n* = 52, 14.6% of all participants
Demographics					
Age (years), median [IQR]	54.0 [48.0, 59.0]	56.0 [51.0, 60.0]	55.5 [49.8, 60.0]	60.5 [58.0, 63.8]	52.5 [45.0, 56.0]
Sex (women), *n* (%)	150 (42.1)	97 (43.5)	30 (39.5)	2 (14.3)	16 (30.8)
Ethnicity, *n* (%)					
Dutch	171 (48.0)	127 (57.0)	46 (60.5)	12 (85.7)	14 (26.9)
South-Asian Surinamese	119 (33.4)	71 (31.8)	18 (23.7)	2 (14.3)	19 (36.5)
Moroccan	66 (18.5)	25 (11.2)	12 (15.8)	0 (0.0)	19 (36.5)
Cardiovascular risk factors					
BMI (kg/m^2^), mean (SD)	25.9 (3.7)	25.9 (3.6)	25.4 (3.6)	26.4 (3.4)	26.2 (3.9)
Smoking, *n* (%)					
Current	84 (23.8)	52 (23.4)	28 (37.3)	1 (7.1)	8 (15.7)
Former	116 (32.9)	92 (41.4)	24 (32.0)	8 (57.1)	10 (19.6)
SBP (mmHg), mean (SD)	131.3 (16.5)	133.6 (17.4)	133.5 (17.7)	144.1 (20.3)	126.8 (13.6)
DBP (mmHg), mean (SD)	81.6 (9.5)	82.1 (9.9)	81.2 (10.2)	84.9 (9.8)	80.8 (9.0)
Hypertension, *n* (%)	143 (40.5)	108 (48.6)	32 (42.1)	10 (71.4)	13 (25.5)
Antihypertensive medication, *n* (%)	68 (19.3)	56 (25.2)	18 (23.7)	4 (28.6)	3 (5.9)
Diabetes mellitus, *n* (%)	35 (9.9)	28 (12.6)	10 (13.2)	1 (7.1)	1 (2.0)
Total cholesterol (mmol/L), mean (SD)	5.4 (1.1)	5.5 (1.2)	5.5 (1.2)	6.1 (1.2)	5.2 (0.8)
LDL (mmol/L), mean (SD)	3.4 (1.0)	3.5 (1.1)	3.6 (1.2)	3.9 (1.1)	3.3 (0.7)
HDL (mmol/l), mean (SD)	1.4 (0.4)	1.5 (0.5)	1.4 (0.4)	1.6 (0.4)	1.3 (0.3)
Lipoprotein(a) (nmol/L), median [IQR]	30.8 [11.5, 86.0]	28.3 [11.6, 83.6]	21.0 [8.4, 79.1]	32.5 [15.4, 124.2]	45.0 [19.1, 95.0]
Lipid-lowering medication, *n* (%)	60 (17.0)	48 (21.6)	16 (21.1)	1 (7.1)	3 (5.9)
CKD, *n* (%)	14 (3.9)	10 (4.5)	4 (5.3)	1 (7.1)	2 (3.8)
Antithrombotic medication, *n* (%)	31 (8.8)	28 (12.6)	8 (10.5)	1 (7.1)	0 (0.0)
History of CVD, *n* (%)	28 (8.0)	25 (11.3)	7 (9.3)	2 (14.3)	1 (0.3)

Participants could exhibit multiple plaque characteristics within a single plaque or have plaques present in both carotid arteries

*IQR* interquartile range, *SD* standard deviation, *BMI* body mass index, *SBP* systolic blood pressure, *DBP* diastolic blood pressure, *eGFR* estimated glomerular filtration rate, *LDL* low-density cholesterol, *LRNC* lipid-rich necrotic core, *HDL* high-density cholesterol, *CKD* chronic kidney disease, *CVD* cardiovascular diseases, *NASCET* North American Symptomatic Carotid Endarterectomy Trial, *IPH* intraplaque haemorrhage

### Plaque characteristics

**Fig. 2:**
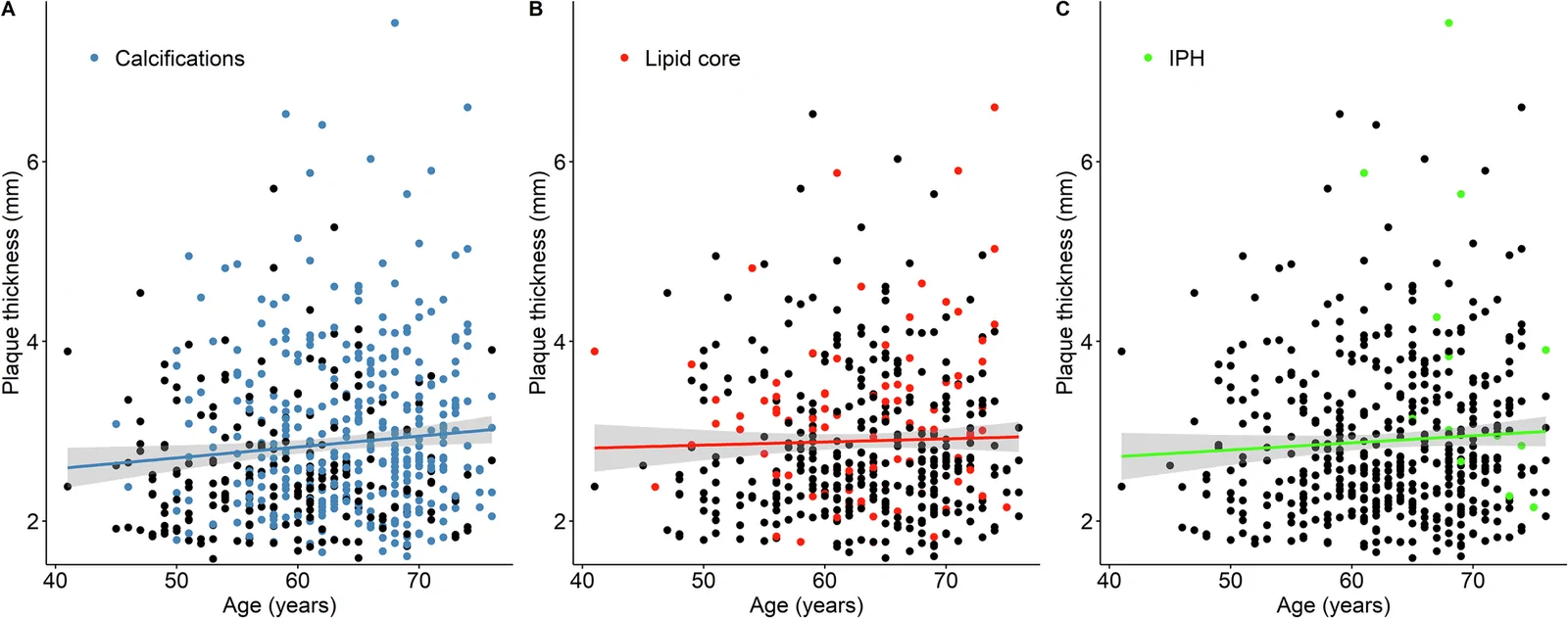
Association between plaque thickness and age, with colour-coded participants indicating the presence of calcification (**A**), LRNC (**B**), and IPH (**C**). IPH, intraplaque haemorrhage; LRNC, lipid-rich necrotic core

Calcification was present in 223 participants (62.6%), a LRNC in 76 participants (21.3%), and IPH in 14 participants (3.9%) (Table 2). The prevalence of significant luminal stenosis was low (5.6% on the left carotid artery, 6.5% on the right carotid artery). Fig. 2 shows the association between plaque thickness and age, with different plaque components represented by the different colours. Calcifications and LRNC were present across all ages and plaque thicknesses, whereas IPH was most common in older participants.

**Table 2:** Plaque characteristics of the included population

	All participants with plaques on one or both sides *n* = 356	Participants with calcifications *n* = 223 62.6% of all participants	Participants with LRNC *n* = 76 21.3% of all participants	Participants with IPH *n* = 14 3.9% of all participants	Participants without plaque characteristics *n* = 52 14.6% of all participants
Plaque location, *n* (%)					
Left side					
Bifurcation	257 (72.8)	163 (74.1)	56 (74.7)	6 (46.2)	38 (73.1)
Common carotid artery	9 (2.5)	7 (3.2)	3 (4.0)	0 (0.0)	0 (0.0)
Internal carotid artery	35 (9.9)	27 (12.3)	11 (14.7)	6 (46.2)	3 (5.8)
Right side					
Bifurcation	245 (69.4)	160 (72.1)	53 (69.7)	10 (71.4)	40 (76.9)
Common carotid artery	6 (1.7)	5 (2.3)	2 (2.6)	1 (7.1)	1 (1.9)
Internal carotid artery	42 (11.9)	36 (16.2)	15 (19.7)	2 (14.3)	1 (1.9)
Plaque thickness (mm), median [IQR]					
Left side	2.9 (0.9)	3.0 (0.9)	2.9 (0.8)	3.7 (1.4)	2.6 (0.5)
Right side	2.8 (0.9)	2.9 (0.9)	3.2 (1.0)	3.4 (1.4)	2.7 (0.8)
Stenosis (NASCET > 30%), *n* (%)					
Left side	20 (5.6)	18 (8.1)	8 (10.5)	2 (14.3)	0 (0.0)
Right side	23 (6.5)	17 (7.6)	15 (19.7)	2 (14.3)	3 (5.8)

Participants could exhibit multiple plaque characteristics within a single plaque or have plaques present in both carotid arteries

*IQR,* interquartile range; NASCET, North American Symptomatic Carotid Endarterectomy Trial; IPH, intraplaque haemorrhage; LRNC, lipid-rich necrotic core

### Demographic factors and plaque components

Age at baseline was significantly associated with calcifications (OR 1.19, 95% CI: 1.10–1.29) and IPH (1.21, 95% CI: 1.02–1.44), but not with LRNC (1.01, 95% CI: 0.97–1.06) (Table 3). No sex differences were observed in the prevalence of any plaque component. There were no significant differences in the prevalence of plaque components between South-Asian Surinamese and the Dutch. In Moroccans, a significantly lower prevalence of calcifications (OR 0.18, 95% CI: 0.05–0.62) was observed compared to the Dutch. Since no Moroccans had plaques with IPH, no estimates could be determined for this characteristic.

**Table 3:** Association between cardiovascular risk factors and the presence of calcifications, LRNC, and IPH

	Calcifications		LRNC		IPH	
	Model 1	Model 2	Model 1	Model 2	Model 1	Model 2
Demographics						
Age (y)	1.19 (1.1; 1.29)^*^	1.13 (1.06; 1.20)^*^	1.01 (0.97; 1.06)	1.01 (0.96; 1.06)	1.21 (1.02; 1.44)^*^	1.24 (1.02; 1.50)^*^
Sex (ref = women)	0.93 (0.44; 1.97)	1.44 (0.69; 3.02)	0.88 (0.48; 1.61)	1.07 (0.55; 2.07)	0.29 (0.06; 1.49)	0.26 (0.04; 1.79)
Ethnicity (ref = Dutch)						
South Asian Surinamese	0.52 (0.22; 1.24)	0.51 (0.21; 1.25)	0.49 (0.24; 1.00)	0.39 (0.17 ; 0.89)^*^	0.10 (0.01; 1.21)	0.05 (0.00; 1.05)
Moroccan	0.18 (0.05 ; 0.62)^*^	0.26 (0.08 ; 0.83)^*^	1.25 (0.51; 3.08)	1.27 (0.45; 3.57)	NA	NA
Risk factors						
BMI (kg/m^2^)	1.04 (0.94; 1.16)	0.96 (0.86; 1.07)	0.98 (0.9; 1.08)	0.98 (0.89; 1.08)	1.06 (0.84; 1.35)	1.01 (0.73; 1.39)
Smoking						
Current	1.56 (0.62; 3.92)	1.29 (0.53; 3.14)	2.88 (1.3 ; 6.40)^*^	2.80 (1.23 ; 6.38)^*^	0.06 (0.00; 1.38)	0.01 (0.00; 0.58)^*^
Former	4.94 (1.79; 13.62)^*^	4.04 (1.57; 10.42)^*^	1.03 (0.48; 2.21)	1.00 (0.45; 2.22)	0.96 (0.20; 4.73)	0.62 (0.10; 3.71)
Diabetes mellitus	6.00 (1.44; 24.97)^*^	2.66 (0.73; 9.73)	1.89 (0.72; 4.98)	1.31 (0.45; 3.80)	NA	NA
Total cholesterol/HDL ratio	1.02 (0.74; 1.41)	1.04 (0.76; 1.43)	1.19 (0.92; 1.53)	1.18 (0.89; 1.55)	1.22 (0.65; 2.27)	0.98 (0.43; 2.23)
Hypertension	4.02 (1.59; 10.12)^*^	2.80 (1.22; 6.42)^*^	1.12 (0.59; 2.13)	1.28 (0.63; 2.58)	1.74 (0.43; 7.13)	2.37 (0.43; 13.18)
CKD	4.36 (0.55; 34.81)	1.82 (0.24; 13.55)	1.20 (0.31; 4.72)	0.44 (0.08; 2.50)	2.91 (0.20; 42.36)	12.14 (0.47; 312.62)
History of CVD	4.66 (1.02; 21.28)^*^	2.47 (0.56; 10.83)	1.22 (0.41; 3.64)	0.86 (0.25; 2.99)	0.59 (0.04; 7.86)	2.17 (0.12; 38.67)

Model 1 was adjusted for age, sex, ethnicity, group of inclusion (0 = reference, 1 = case), follow-up time, and maximum wall thickness. Model 2 was additionally adjusted for other cardiovascular risk factors. In the IPH model, some variables have missing values (NA) because the number of participants with those specific characteristics is too low to estimate reliable coefficients

*BMI* body mass index, *HDL* high-density lipoprotein, *CKD* chronic kidney disease, *CVD* cardiovascular diseases, *IPH* intraplaque haemorrhage, *LRNC* lipid-rich necrotic core

^*^Indicates a significant association (*p* < 0.05)

### Cardiovascular risk factors and calcification

Compared to non-smokers, former smokers had a significantly higher prevalence of calcifications (OR 4.94, 95% CI: 1.79–13.62), with similar estimates after adjustment for cardiovascular risk factors (model 2). Diabetes mellitus was associated with calcification (OR 6.00, 95% CI: 1.44–24.97), but not after adjustment for other cardiovascular risk factors (model 2, OR 2.66, 95% CI: 0.73–9.73). Hypertension was positively associated with calcification (OR 4.02, 95% CI: 1.59–10.12), with similar results after adjusting for cardiovascular risk factors (model 2). A history of CVD was associated with the presence of calcifications (OR 4.66, 95% CI: 1.02–21.28), but this effect was attenuated after further adjustments (model 2). No associations were found for BMI, total cholesterol/HDL ratio, and CKD. We did find an association between the use of antithrombotic medication, lipid-lowering medication and antihypertensive medication. However, these effects disappeared after further adjustment for other cardiovascular risk factors (Supplementary Table 4).

### Cardiovascular risk factors and LRNC

For LRNC, smoking was associated with an increased prevalence of lipid (OR 2.88, 95% CI: 1.3–6.4), which was also significantly associated after further adjustment for cardiovascular risk factors (model 2, OR 2.80, 95% CI: 1.23–6.38). No associations were found with BMI, diabetes, total cholesterol/HDL ratio, hypertension, CKD and history of CVD.

### Cardiovascular risk factors and IPH

In the regression models for IPH, some variables had missing values (NA) due to the low prevalence of IPH (*n* = 14). We found no significant associations between cardiovascular risk factors and IPH, although the analysis was limited by low statistical power.

### Sensitivity analyses

Excluding participants with a history of CVD yielded similar results (Supplementary Table 2). Combining plaque characteristics into a composite variable showed no association with cardiovascular risk factors (Supplementary Table 3), although South-Asian Surinamese had a lower prevalence compared to the Dutch (OR 0.42, 95% CI: 0.19–0.92). Antithrombotic medication use was not associated with LRNC or IPH (data not shown), while a significant association with calcifications (OR 5.04, 95% CI: 1.16–21.88) was observed, but this disappeared after adjustment for cardiovascular risk factors. Replacing hypertension with SBP revealed a modest association (OR 1.03, 95% CI: 1.00–1.05), while no association was found with DBP. Substituting the total cholesterol/HDL ratio with corrected LDL values produced similar results (data not shown).

## Discussion

In this multi-ethnic population with carotid plaques, we examined associations between cardiovascular risk factors and plaque components using 3-T MRI after 8.4 years of follow-up. Calcifications were most common, followed by LRNC, while IPH was rare. Calcifications were associated with smoking and hypertension, while associations with diabetes and CVD history were not significant after adjustment. For LRNC, we found an association with smoking, while no cardiovascular risk factors were associated with IPH. However, given the low prevalence of IPH in this cohort (*n* = 14, 3.9%), the statistical power for these analyses is limited, and the IPH-related findings should therefore be interpreted with caution and considered exploratory. The findings of our study suggest that adverse midlife cardiovascular profiles are related to specific plaque components later in life, highlighting the importance of considering plaque characteristics individually in CVD risk assessment [5]. Our study adds new insights by focusing on a midlife population with a long interval between risk factor assessment and MRI assessment. These design elements extend prior findings and emphasise the relevance of early-life cardiovascular risk management for later-life plaque composition.

Consistent with a previous study involving younger participants from the general population, we observed a low prevalence of IPH [20], while population-based studies that included older individuals reported a higher prevalence of IPH [8, 12]. It is important to note that the MRI cohort was derived from a subpopulation enriched based on carotid ultrasound findings, which may have influenced the observed prevalence of plaque components and should be considered when comparing our results with fully population-based cohorts. Additionally, methodological differences in MRI protocols could play a role, as we used the SNAP sequence to detect IPH, which could lead to either an overestimation or underestimation of its prevalence. Previous studies in patients with symptomatic carotid plaques have shown strong relationships between IPH and recurrent stroke [4, 21, 22], raising the question of whether screening for IPH to identify high-risk patients could be beneficial. However, in our study examining a midlife population, the prevalence of IPH was very low, despite the oversampling of participants with carotid plaques on US.

Our study found lower calcification prevalence in Moroccans compared to the Dutch, consistent with their lower stroke risk [23]. In South-Asian Surinamese, despite a higher expected risk, plaque component prevalence was also lower than in the Dutch, aligning with previous findings of lower carotid plaque and IMT in this group [14]. Possible explanations include selection bias due to the inclusion of healthier participants in this sub-study or the possibility that South-Asian Surinamese may have a higher stroke risk due to non-ischaemic factors.

Other population-based studies exploring cardiovascular risk factors and carotid plaque components include the Rotterdam Study, MESA, ARIC, and CHAMPS [8–11]. The Rotterdam Study (1,006 participants of European descent, mean age ~70 years), also evaluated calcifications, LRNC, and IPH separately [8]. Consistent with our findings, they found associations of smoking and hypertension with calcifications.

In our study, no significant association was found between total cholesterol/HDL ratio and LRNC, in contrast to the MESA study (214 participants, mean age of 66.7 years) and the Rotterdam Study [8, 10], while in the ARIC Study, which assessed risk factors at baseline (18 years before imaging) and at the time of MRI, LDL was associated with LRNC, though this association was only evident in models unadjusted for wall thickness [9]. In the CHAMPS study, a strong relation was found between the cumulative burden of risk factors at baseline and the presence of LRNC (424 participants, mean age 73 years at the time of MRI) [11]. Discrepancies between our findings and others may be attributed to differences in study populations and imaging techniques. Additionally, MRI techniques, such as gadolinium-enhanced MRI, used in other studies, improve LRNC detection [24]. Our use of the SNAP sequence, which differentiates LRNC from IPH, likely reduced bias, as other studies did not separate these components, potentially conflating results, as IPH frequently coexists with LRNC [25, 26].

Strengths of this study include the use of a 3-T MRI, enabling us to increase the resolution of the MRI images [27]. Furthermore, we included mid-life, whereas the mean age in other studies was around 70 years, limiting their applicability to younger populations. Limitations of this study include the absence of gadolinium contrast-enhanced MRI sequences, which were not feasible on a large scale in a population-based setting. This made determining LRNC more difficult, as evidenced by the modest interobserver kappa statistics for LRNC, potentially leading to underestimation of prevalence and attenuation of the observed associations with cardiovascular risk factors. Furthermore, due to the lack of contrast-enhanced MRI in our study, we could not focus on other plaque characteristics, such as the presence of a fibrous cap. Moreover, the low prevalence of IPH considerably limited the statistical power of the analysis. Therefore, the absence of significant associations with IPH should be interpreted with caution and not be considered evidence of the absence of a true relationship. Finally, the median interval of 8.4 years between risk factor assessment and MRI may also have introduced variability due to changes in cardiovascular risk factors during follow-up, potentially leading to some misclassification and attenuation of observed associations. Although risk factor data from approximately two years prior to MRI were available, we did not use these, as our primary focus was on the long-term impact of midlife cardiovascular risk factors on later-life plaque composition.

Our findings support the hypothesis that midlife cardiovascular risk factors, even in the absence of overt cardiovascular disease (CVD), could contribute to increased risk later in life. The use of carotid plaque characterisation as a marker of subclinical atherosclerotic disease in the general population may be limited, but it could be of added value in individuals with a high risk of CVD, given the higher prevalence of plaque features in this group and the opportunities for intervention to reduce cardiovascular risk. Future studies should include larger sample sizes to increase statistical power and enable more detailed quantitative analyses of plaque characteristics. Furthermore, additional imaging modalities and outcomes, such as carotid CT imaging, fibrous cap assessment, perivascular adipose tissue, and luminal geometry, warrant further investigation [28, 29].

## Supplementary information

ELECTRONIC SUPPLEMENTARY MATERIAL

## Abbreviations

BB: Black blood
BMI: Body mass index
BP: Blood pressure
cIMT: Carotid intima-media thickness
CKD: Chronic kidney disease
CVD: Cardiovascular disease
HDL: High-density lipoprotein
ICC: Intraclass correlation coefficient
IPH: Intraplaque haemorrhage
IQR: Interquartile range
LDL: Low-density lipoprotein
LRNC: Lipid-rich necrotic core
MRI: Magnetic resonance imaging
NASCET: North American Symptomatic Carotid Endarterectomy Trial
SD: Standard deviation
SNAP: Simultaneous non-contrast angiography and intraplaque haemorrhage
TE: Echo time
TR: Repetition time
US: Ultrasonography
