# Supplementary material for: Cardiovascular risk factors and carotid plaque components in a multi-ethnic cohort using 3 Tesla MRI: the HELIUS study
Source: Eur Radiol. 2026 Jun 27;36(11):8277–86. doi: 10.1007/s00330-026-12713-2 (PMC13661681; doi:10.1007/s00330-026-12713-2)
Supplement: Supplementary file 1 [file 330_2026_12713_MOESM1_ESM.pdf]

Cardiovascular risk factors and carotid plaque components in a multi-ethnic cohort using 3 Tesla MRI: The HELIUS study

ELECTRONIC SUPPLEMENTARY MATERIAL

Eur Radiol (2026) Vriend EMC, Bouwmeester TA, Bos D et al.

Supplementary Figure 1: Study design. Ten percent of participants from the ultrasonography sub-study were randomly chosen for the MRI visit, regardless of US results, as a representative sample of the general population (reference group, n = 300). The remaining participants were invited based on the presence of atherosclerosis on carotid US (case group, n = 271).

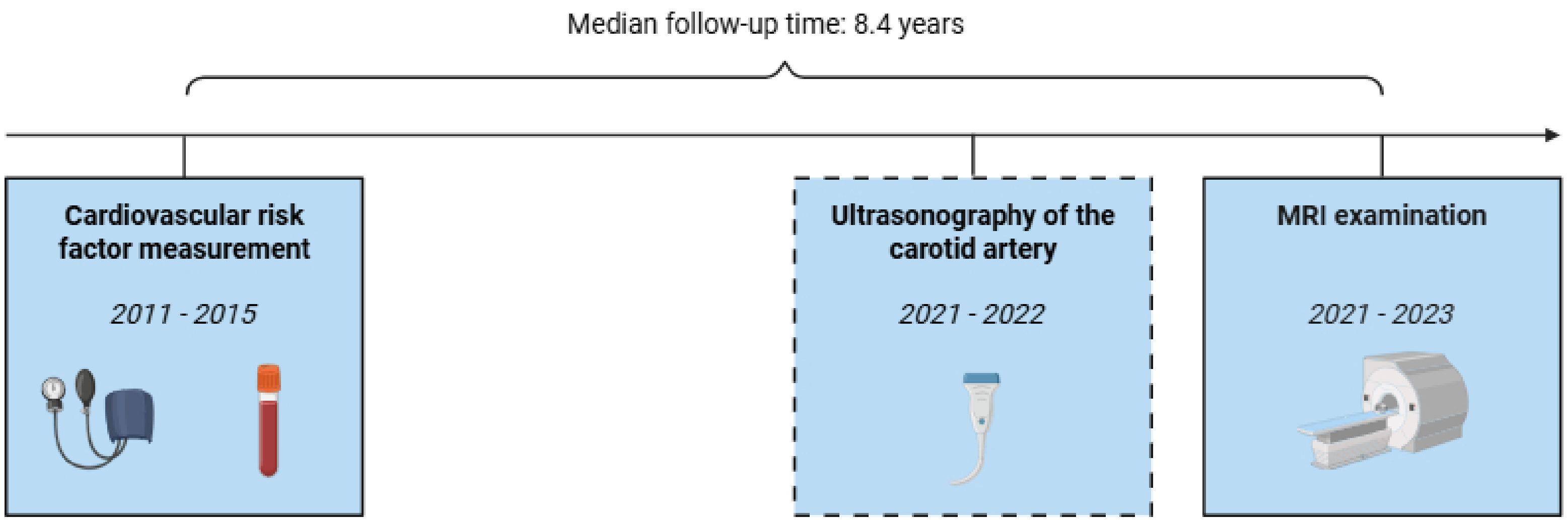

MRI = magnetic resonance imaging.

Supplementary Figure 2: Overview of the selection of participants.

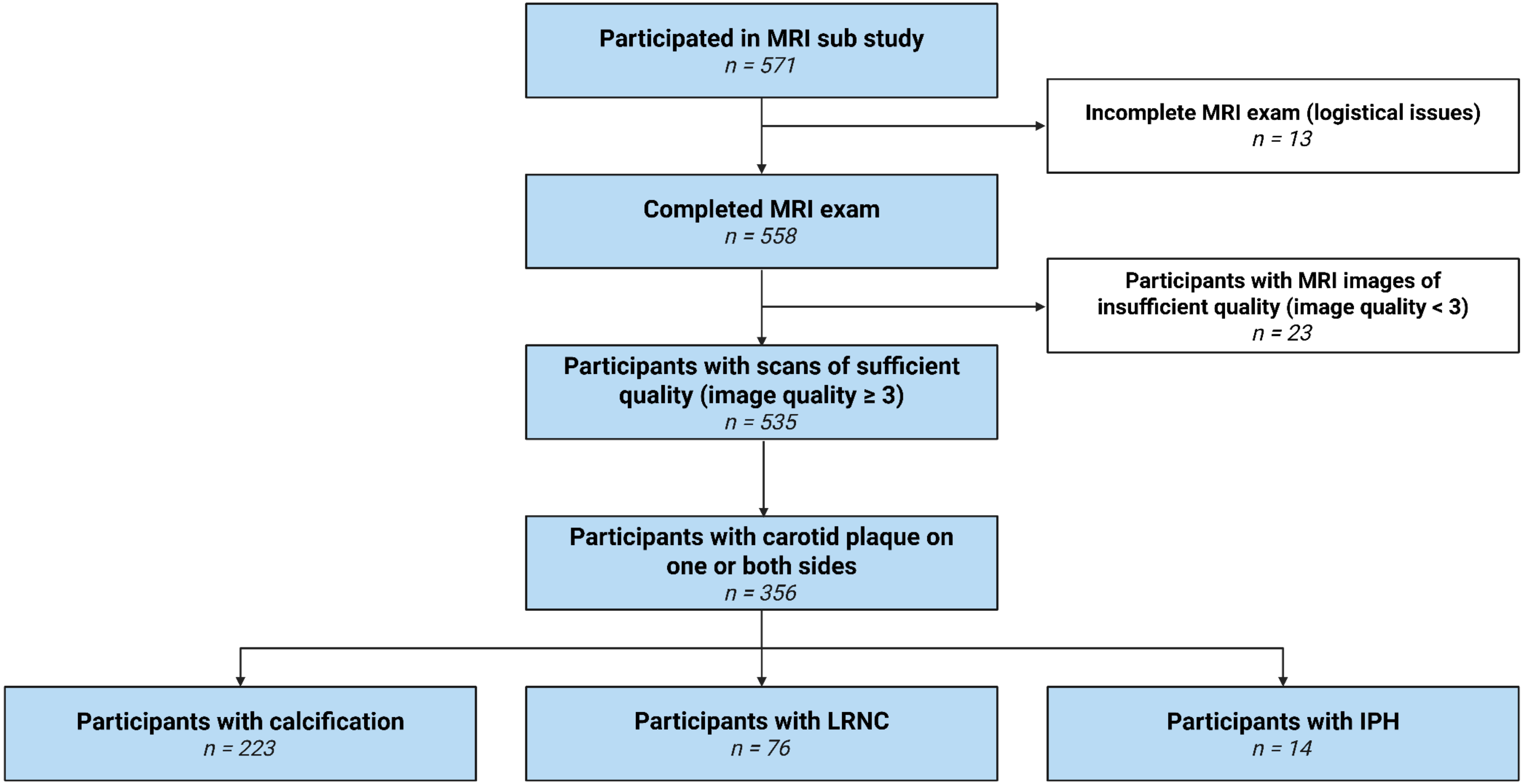

MRI = magnetic resonance imaging. IPH = intraplaque haemorrhage. LRNC = lipid rich necrotic core.

Supplementary Figure 3: Histogram of follow-up time with cumulative distribution of participants.

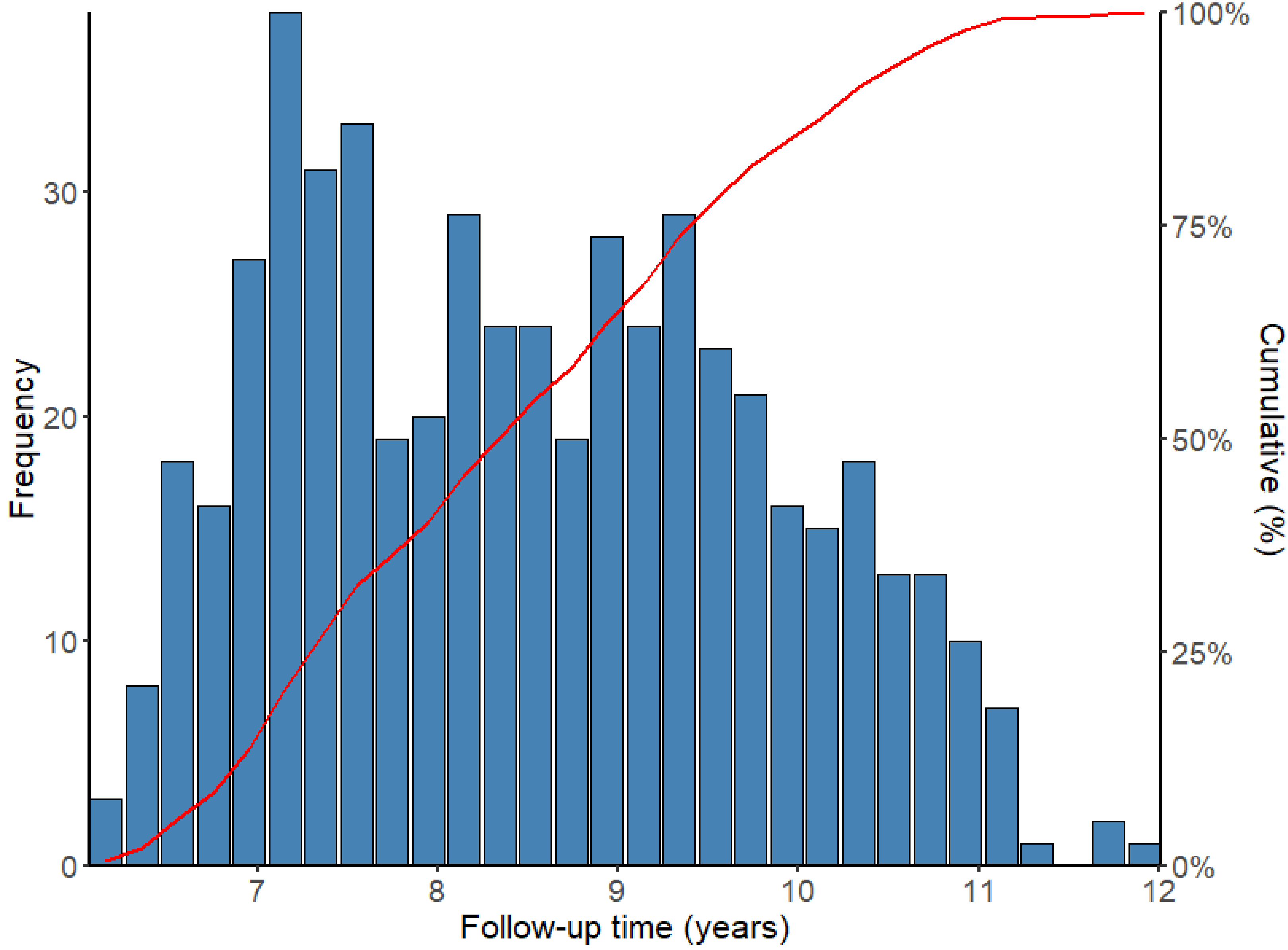

Supplementary Table 1: MRI image acquisition parameters.

	**T1-BB (iMSDE)**	**T2-BB**	**SNAP**
TR (ms)	10	3000	10
TE (ms)	3.6	80	5
Flip angle (°)	6	90	11
FOV (mm)			
FH	156	150	156
RL	156	150	156
AP	35	27	35.2
Acquired voxel size (mm^3)	0.50x0.50x0.50	0.55x0.76x3.00	0.80x0.80x1.60
Reconstructed voxel size (mm^3)	0.49x0.49x0.50	0.39x0.39x3.00	0.39x0.39x0.80

BB = black blood. Improved Motion-Sensitized Driven-Equilibrium (iMSDE). SNAP = Simultaneous Non-contrast Angiography. TR = repetition time. TE = echo time. FOV = field-of-view.

Supplementary Table 2: Association between cardiovascular risk factors and the presence of calcifications, lipid-rich necrotic core, and intraplaque haemorrhage after excluding participants with a history of CVD. Model 1 was adjusted for age, sex, ethnicity, group of inclusion (0 = reference, 1 = case), follow-up time, and maximum wall thickness. Model 2 was additionally adjusted for other cardiovascular risk factors. In the IPH model, some variables have missing values (NA) because the number of

	**Calcifications**		**LRNC**		**IPH**	
	Model 1	Model 2	Model 1	Model 2	Model 1	Model 2
**Demographics**						
Age (y)	1.17 (1.09 ; 1.27)*	1.13 (1.05 ; 1.21)*	1.00 (0.96 ; 1.05)	1.01 (0.96 ; 1.06)	1.21 (1.02 ; 1.44)*	1.21 (1 ; 1.46)*
Sex (Ref = women)	1.02 (0.47 ; 2.20)	1.35 (0.61 ; 2.96)	0.92 (0.50 ; 1.71)	1.00 (0.52 ; 1.93)	0.29 (0.05 ; 1.58)	0.26 (0.04 ; 1.75)
Ethnicity (Ref = Dutch)						
South Asian Surinamese	0.48 (0.19 ; 1.20)	0.58 (0.22 ; 1.53)	0.47 (0.22 ; 1.01)	0.39 (0.17 ; 0.91)*	0.24 (0.02 ; 2.49)	0.14 (0.01 ; 2.05)
Moroccan	0.2 (0.06 ; 0.72)*	0.25 (0.07 ; 0.89)*	1.1 (0.44 ; 2.76)	1.11 (0.40 ; 3.08)	NA	NA
**Risk factors**						
BMI (kg/m^2)	1.05 (0.94 ; 1.17)	0.98 (0.88 ; 1.10)	1.01 (0.92 ; 1.10)	1.00 (0.91 ; 1.11)	1.06 (0.83 ; 1.36)	1.10 (0.79 ; 1.53)
Smoking						
Current	1.56 (0.59 ; 4.15)	1.47 (0.55 ; 3.95)	2.31 (1.02 ; 5.23)*	2.27 (0.99 ; 5.19)	0.11 (0.00 ; 2.93)	0.03 (0.00 ; 1.1)
Former	4.74 (1.66 ;	4.07 (1.44 ;	0.95 (0.43 ; 2.08)	0.91 (0.40 ; 2.03)	0.85 (0.16 ; 4.45)	0.46 (0.07 ; 2.89)
Diabetes mellitus	7.28 (1.44 ;	4.47 (0.97 ; 20.65)	1.48 (0.51 ; 4.26)	1.35 (0.46 ; 3.98)	NA	NA
Total cholesterol/HDL ratio	1.01 (0.73 ; 1.41)	0.99 (0.70 ; 1.39)	1.17 (0.90 ; 1.53)	1.14 (0.87 ; 1.51)	0.9 (0.45 ; 1.8)	0.62 (0.22 ; 1.72)
Hypertension	3.67 (1.42 ; 9.44)*	2.90 (1.18 ; 7.12)*	1.18 (0.62 ; 2.28)	1.27 (0.63 ; 2.55)	1.84 (0.42 ; 8.13)	2.58 (0.46 ; 14.43)
CKD	3.1 (0.32 ; 30.21)	2.13 (0.21 ; 21.17)	0.74 (0.15 ; 3.62)	0.46 (0.08 ; 2.57)	3.3 (0.22 ; 50.27)	13.5 (0.54 ;

participants with those specific characteristics is too low to estimate reliable coefficients. * Indicates a significant association ($p<0.05$).

BMI = body mass index. CKD = chronic kidney disease. CVD = cardiovascular diseases. IPH = intraplaque haemorrhage. LRNC = lipid rich necrotic core.

Supplementary Table 3: Association between cardiovascular risk factors and having at least two plaque characteristics (calcifications, lipid-rich necrotic core and/or intraplaque haemorrhage). Model 1 was adjusted for age, sex, ethnicity, group of inclusion (0 = reference, 1 = case), follow-up time, and maximum wall thickness. Model 2 was additionally adjusted for other cardiovascular risk factors. * Indicates a significant association ($p<0.05$).

	**Combination of two or three plaque characteristics**	
	Model 1	Model 2
**Demographics**		
Age (y)	1.04 (0.99 ; 1.09)	1.03 (0.97 ; 1.09)
Sex (Ref = women)	0.74 (0.39 ; 1.41)	0.88 (0.44 ; 1.77)
Ethnicity (Ref = Dutch)		
South Asian Surinamese	0.42 (0.19 ; 0.92)*	0.39 (0.16 ; 0.98)*
Moroccan	0.69 (0.26 ; 1.84)	0.81 (0.27 ; 2.44)
**Risk factors**		
BMI (kg/m^2)	0.97 (0.88 ; 1.07)	0.95 (0.85 ; 1.06)
Smoking		
Current	2.25 (0.99 ; 5.13)	2 (0.85 ; 4.70)
Former	1.58 (0.68 ; 3.63)	1.57 (0.66 ; 3.72)
Diabetes mellitus	1.27 (0.46 ; 3.47)	0.78 (0.24 ; 2.48)
Total cholesterol/HDL ratio	1.11 (0.85 ; 1.46)	1.11 (0.82 ; 1.50)
Hypertension	1.33 (0.68 ; 2.61)	1.43 (0.69 ; 2.96)
CKD	2.02 (0.51 ; 8.07)	1.29 (0.25 ; 6.60)
History of CVD	1.25 (0.41 ; 3.87)	1 (0.28 ; 3.57)

BMI = body mass index. CKD = chronic kidney disease. CVD = cardiovascular diseases.

Supplementary Table 4: Association between use of antithrombotic medication, lipid lowering medication, antihypertensive medication and the presence of calcifications, lipid-rich necrotic core, and intraplaque haemorrhage. Model 1 was adjusted for age, sex, ethnicity, group of inclusion (0 = reference, 1 = case), follow-up time, and maximum wall thickness. Model 2 was additionally adjusted for other cardiovascular risk factors. In the IPH model, some variables have missing values (NA) because the number of participants with those specific characteristics is too low to estimate reliable coefficients. * Indicates a significant association (p<0.05).

	**Calcifications**		**LRNC**		**IPH**	
	Model 1	Model 2	Model 1	Model 2	Model 1	Model 2
Use of medication						
Antithrombotic medication	5.04 (1.16 ;	2.23 (0.34 ; 14.63)	1.05 (0.38 ; 2.9)	0.76 (0.16 ; 3.59)	0.3 (0.03 ; 3.46)	0.07 (0 ; 22.72)
Lipid lowering medication	4.51 (1.49 ;	2.12 (0.67 ; 6.74)	1.46 (0.65 ; 3.29)	2.12 (0.67 ; 6.74)	0.28 (0.03 ; 3.07)	0.4 (0.03 ;
Antihypertensive medication	6.29 (1.97 ;	5.1 (1.58 ; 16.4)*	1.85 (0.85 ; 4.03)	1.7 (0.69 ; 4.2)	0.93 (0.18 ; 4.91)	2.11 (0.26 ; 17.13)

CVD = cardiovascular diseases. IPH = intraplaque haemorrhage. LRNC = lipid rich necrotic core.
